# Correction: Transcriptional analysis of long non-coding RNAs in facet joint osteoarthritis

**DOI:** 10.1039/c8ra90079e

**Published:** 2018-10-18

**Authors:** Chu Chen, Guanhua Xu, Kun Yuan, Yuyu Sun, Guofeng Bao, Dawei Xu, Zhiming Cui

**Affiliations:** Department of Spine Surgery, The Second Affiliated Hospital of Nantong University 6 Hai’er Alley North, Chongchuan District Nantong 226001 Jiangsu China czmspine@126.com

## Abstract

Correction for ‘Transcriptional analysis of long non-coding RNAs in facet joint osteoarthritis’ by Chu Chen *et al.*, *RSC Adv.*, 2018, **8**, 33695–33701.

The authors regret that a footnote to indicate that Chu Chen and Guanhua Xu are co-first authors was omitted in the original manuscript.

The Royal Society of Chemistry apologises for these errors and any consequent inconvenience to authors and readers.

## Supplementary Material

